# Phase 1 dose escalation study of seribantumab (MM-121), an anti-HER3 monoclonal antibody, in patients with advanced solid tumors

**DOI:** 10.1007/s10637-021-01145-y

**Published:** 2021-07-11

**Authors:** Crystal S. Denlinger, Vicki L. Keedy, Victor Moyo, Gavin MacBeath, Geoffrey I. Shapiro

**Affiliations:** 1 Department of Hematology/Oncology, Fox Chase Cancer Center, Philadelphia, PA USA; 2 Department of Medicine (Hematology and Oncology), Vanderbilt-Ingram Cancer Center, Nashville, TN USA; 3 Merrimack Pharmaceuticals, Inc., Cambridge, MA USA; 4 Dana-Farber Cancer Institute, Boston, MA USA

**Keywords:** Seribantumab, MM-121, HER3, Monoclonal antibody, Anti-HER3 mAb, Advanced solid tumor, Dose-escalation study

## Abstract

*Background* Overactivation of human epidermal growth factor receptor 3 (HER3) triggers multiple intracellular pathways resulting in tumor cell survival. This Phase 1 study assessed the safety, efficacy, and pharmacokinetics (PK) of seribantumab, a fully human anti-HER3 monoclonal antibody. *Methods* Adult patients with advanced or refractory solid tumors were treated in six dose cohorts of seribantumab: 3.2, 6, 10, 15, or 20 mg/kg weekly, or 40 mg/kg loading dose followed by 20 mg/kg weekly maintenance dose (40/20 mg/kg) using a modified 3 + 3 dose escalation strategy with cohort expansion. Primary objectives were identification of a recommended Phase 2 dose (RP2D) and determination of objective response rate. Secondary objectives were assessment of safety, dose-limiting toxicities, and PK. *Results* Forty-four patients (26 dose escalation; 18 dose expansion) were enrolled. Seribantumab monotherapy was well tolerated with most adverse events being transient and mild to moderate (grade 1 or 2) in severity; maximum tolerated dose was not reached. The highest dose, 40/20 mg/kg, was identified as RP2D. Best response was stable disease, reported in 24% and 39% of patients during the dose escalation and expansion portions of the study, respectively. Seribantumab terminal half-life was ≈100 h; steady state concentrations were reached after 3–4 weekly doses. *Conclusions* Seribantumab monotherapy was well tolerated across all dose levels. Safety and PK data from this study support further seribantumab investigations in genomically defined populations.

*Clinical trial registration *NCT00734305. August 12, 2008.

## Introduction

The human epidermal growth factor receptor (HER; ERBB) family includes four transmembrane receptor tyrosine kinases: epidermal growth factor receptor (ERBB1), HER2 (ERBB2), HER3 (ERBB3), and HER4 (ERBB4) [1]. Homo- or heterodimerization between members of the ERBB family leads to activation of downstream signaling pathways mediating tumor growth, survival, and differentiation [2, 3]. The most potent dimerization occurs between HER2 and HER3, which results in enhanced signal transduction compared with other ERBB family dimers [4]. Elevated expression of HER3 is seen in many solid tumors, including lung, colorectal, breast, and ovarian cancers, and is typically associated with poor prognosis [4–7].

Seribantumab (formerly MM-121; SAR256212), a fully human immunoglobulin (Ig) G2 anti-HER3 monoclonal antibody, was initially developed for the treatment of solid tumors in which HER3 signaling is active [8]. In recent in vitro studies, seribantumab has been shown to block ligand-dependent activation of HER3, HER2-HER3 dimerization, and, to a lesser extent, dimerization with other ERBB family members [9]. Of note, in tumor models harboring NRG1 fusions, seribantumab leads to reduced phosphorylation across the ERBB family and subsequent inhibition of downstream signaling pathways, including the phosphoinositide 3-kinase protein kinase B and mitogen-activated protein kinase pathways, resulting in the inhibition of cancer cell survival [9].

Here we present the safety, efficacy, and pharmacokinetic (PK) outcomes from a Phase 1 study of seribantumab monotherapy in patients with solid tumors (NCT00734305), which provide a foundation for subsequent seribantumab studies.

## Methods

### Study design and treatment

This was an open-label, Phase 1, dose escalation and dose expansion study of seribantumab in adult patients with advanced or refractory solid tumors. The study was active from July 2008 (first patient enrolled) to April 2012 (last patient completed).

The study used a modified 3 + 3 dose escalation design, with three to four patients in each cohort. Cohorts were expanded to six patients if at least one patient experienced a dose limiting toxicity (DLT). Seribantumab was administered weekly in 4-week cycles with no pre-medications. A total of six dose cohorts were explored: 3.2, 6, 10, 15, and 20 mg/kg weekly, and a 40 mg/kg loading dose followed by 20 mg/kg weekly maintenance dose (40/20 mg/kg). In each cohort, the first dose was administered over 90 min, with subsequent doses administered over 60 min if no infusion reactions were observed after the first dose. DLTs were defined as drug-related grade 3 or 4 hematologic or non-hematologic toxicities, including grade 3 or 4 infusion reactions, occurring during the first 4 weeks of treatment. Patients continued treatment until disease progression (assessed at Week 4 of alternate cycles) or intolerable toxicity. Patients enrolled in the dose expansion portion of the study received a dose selected based on safety and tolerability in the dose escalation portion of the study, to confirm the recommended Phase 2 dose (RP2D).

### Eligibility criteria

Adult patients (aged ≥ 18 years) with advanced, refractory solid tumors, Eastern Cooperative Oncology Group performance status of 0‒2 and adequate end organ function were eligible to enroll in the dose escalation portion of the study. For the dose expansion cohort, additional eligibility criteria were advanced or metastatic triple-negative breast cancer, advanced or metastatic hormone receptor-positive HER2-negative breast cancer, or advanced or metastatic epithelial ovarian, fallopian tube, or primary peritoneal cancer. Patients with other tumor types, including metastatic colorectal cancer and non-small cell lung cancer, were evaluated for eligibility on a per-patient basis. Patients for whom potentially curative antineoplastic therapy was available, patients who had received standard chemotherapy or radiation within 14 days or investigational therapy within 30 days of study entry, and patients with untreated or symptomatic primary or metastatic central nervous system tumors were excluded from the study.

### Study objectives

The primary objectives of the study were definition of the RP2D and determination of objective response rate per Response Evaluation Criteria in Solid Tumours version 1.0 (RECIST v1.0), including clinical benefit rate. Objective response rate was defined as the proportion of patients with best overall response of complete response (CR) or partial response (PR). Response (CR or PR) was confirmed if two separate consecutive disease assessments showed tumor response. Clinical benefit rate was defined as proportion of patients who achieved overall response of confirmed CR, PR, or stable disease (SD) for at least 16 weeks from the date of tumor assessment up to and including the end of the study. Duration of response was defined as the time from first documented response until subsequently documented progressive disease or death. Secondary objectives included assessment of safety, incidence of DLTs, PK parameters, and progression-free survival.

Treatment-emergent adverse events (TEAEs) were categorized by System Organ Class and Preferred Term using Medical Dictionary for Regulatory Activities version 13.0. Common Terminology Criteria for Adverse Events version 4.0 was used to categorize the severity of adverse events (AEs) as grade 1 to 5. All analyses were produced using Statistical Analysis System (SAS) version 9.2 or higher.

### Pharmacokinetic analysis

Blood samples for PK analysis were collected prior to treatment for each cycle, at the end of Cycles 1 and 2, and then 2, 4, 8, 24, 48, and 72 h after the start of the first and second cycle of seribantumab infusion. Patient serum samples were analyzed at Charles River Laboratories Preclinical Services, Senneville, QC, Canada, using a validated enzyme-linked immunosorbent assay.

### Statistical methods

Safety and efficacy analyses were completed on the safety population, defined as all patients who received at least one seribantumab infusion. The total number of patients enrolled in the dose escalation portion was dependent on the number of cohorts required to identify the maximum tolerated dose (MTD). When a DLT was experienced at a given dose, the cohort was expanded to six patients. The dose escalation plan provided a 91% probability that dose escalation could proceed with doses associated with a DLT probability of < 10%. In the dose expansion portion, a sample size of 30 patients allowed identification of cumulative toxicity at the MTD or RP2D.

## Results

### Patients

A total of 44 patients were enrolled in the study, 26 patients in the dose escalation portion and 18 patients in the dose expansion portion. One patient in the dose escalation portion did not receive seribantumab and was excluded from the analysis (Table 1). Among the 43 patients who received at least one dose of seribantumab (safety population), 63% were female and the most common diagnoses (> 10% of patients) were colorectal (30%), breast (21%), and ovarian (12%) cancer (Table 2). Median age was 62.0 (range: 29–81) years in the dose escalation portion and 57.0 (range: 47–71) years in the dose expansion portion. All patients had received at least one prior systemic therapy related to their cancer diagnosis; 91% (39/43) reported at least three and 59% (24/43) reported at least six prior lines of systemic therapy. The rate of treatment discontinuation due to TEAEs was 8% (2/25) in the dose escalation portion, whereas no patients in the dose expansion portion discontinued due to TEAEs. Overall, 18% (8/43) patients died during the study, with the most common cause being disease progression. No deaths were considered related to seribantumab.

**Table 1:** Patient disposition

	Dose, mg/kg	*n*	Reason for withdrawal
Dose escalation (*n* = 26)	Not dosed	1	Other (patient enrolled but not included in the safety population)
	3.2	6	PD (*n* = 2), AE (*n* = 2), symptomatic deterioration (*n* = 2)
	6	3	PD (*n* = 2), death (*n* = 1)
	10	4	PD (*n* = 3), symptomatic deterioration (*n* = 1)
	15	3	PD (*n* = 2), withdrew consent (*n* = 1)
	20	5	PD (*n* = 2), withdrew consent (*n* = 1), symptomatic deterioration (*n* = 2)
	40/20	4	PD (*n* = 3), symptomatic deterioration (*n* = 1)
Dose expansion (*n* = 18)	40/20	18	PD (*n* = 14), investigator decision (*n* = 1), symptomatic deterioration (*n* = 3)

*40/20* 40 mg/kg loading dose followed by 20 mg/kg maintenance dose, *AE* adverse event, *PD* progressive disease

**Table 2:** Demographics and baseline characteristics in the safety population

Characteristic	Dose escalation (*n* = 25)	Dose expansion (*n* = 18)
Median age, years (range)	62 (29‒81)	57 (47‒71)
Median body weight, kg (range)	75 (44‒119)^a^	71 (55‒140)
Sex, *n* (%)		
Female	12 (48)	15 (83)
Tumor type, *n* (%)		
Colorectal cancer^b^	10 (40)	3 (17)
Breast cancer	1 (4)	8 (44)
Ovarian cancer^c^	1 (4)	4 (22)
Lung cancer	3 (12)	1 (6)
Bladder cancer	0	1 (6)
Melanoma	2 (8)	0
Squamous cell carcinoma^d^	2 (8)	0
Pancreatic cancer	1 (4)	0
Esophageal cancer	1 (4)	0
Mucoepidermoid carcinoma^e^	1 (4)	0
Peritoneal cancer	1 (4)	0
Prostate cancer	1 (4)	0
Thymic carcinoma	1 (4)	0
Urethra carcinoma	0	1 (6)

^a^*n* = 24

^b^Colorectal cancer includes colon, colorectal, rectal, and cecum cancers

^c^Ovarian cancer includes ovarian, fallopian tube, and peritoneal cancers

^d^Left hip (n = 1), site not specified (n = 1)

^e^Primary site not specified

### Safety

#### Dose escalation

In the dose escalation portion of the study, median duration of exposure to seribantumab was 6 (range, 1‒23) weeks. All patients experienced at least one TEAE and the rate of grade ≥ 3 TEAEs was 48% (12/25). All-cause TEAEs reported in ≥ 10% of patients are summarized in Table 3. At least one TEAE related to seribantumab occurred in 84% (21/25) of patients. The most common TEAEs related to seribantumab were nausea (44%), diarrhea (36%), fatigue (28%), and skin rash (24%). No infusion-related reactions or dose-dependency were observed.

**Table 3:** All-cause TEAEs occurring in ≥ 10% of patients in the safety population in either study portion

TEAE, *n* (%)	Dose escalation (*n* = 25)		Dose expansion (*n* = 18)	
	All grades	Grade ≥ 3	All grades	Grade ≥ 3
At least one TEAE	25 (100)	12 (48)	18 (100)	9 (50)
Nausea	12 (48)	0	8 (44)	1 (6)
Diarrhea	10 (40)	0	6 (33)	2 (11)
Fatigue	10 (40)	3 (12)	11 (61)	2 (11)
Anemia^a^	9 (36)	1 (4)	6 (33)	0
Rash	8 (32)	0	2 (11)	0
Decreased appetite	7 (28)	0	5 (28)	0
Hyperglycemia	7 (28)	0	3 (17)	0
Vomiting	6 (24)	0	6 (33)	1 (6)
Hypokalemia	6 (24)	1 (4)	5 (28)	1 (6)
Hypoalbuminemia	6 (24)	0	2 (11)	0
Hypomagnesemia	6 (24)	0	1 (6)	0
Back pain	6 (24)	0	0	0
Hypocalcemia	5 (20)	0	1 (6)	0
Increased alkaline phosphatase	5 (20)	0	0	0
Abdominal pain	4 (16)	1 (4)	3 (17)	0
Dehydration	4 (16)	1 (4)	3 (17)	1 (6)
Dyspepsia	4 (16)	0	1 (6)	0
Increased AST	4 (16)	0	0	0
Decreased weight	3 (12)	0	6 (33)	0
Dyspnea	3 (12)	1 (4)	4 (22)	2 (11)
Dysuria	3 (12)	0	2 (11)	0
Hyponatremia	3 (12)	1 (4)	1 (6)	0
Constipation	3 (12)	0	1 (6)	0
Stomatitis	3 (12)	0	1 (6)	0
Disease progression	3 (12)	3 (12)	1 (6)	1 (6)
Edema peripheral	3 (12)	1 (4)	1 (6)	1 (6)
Dizziness	3 (12)	0	1 (6)	1 (6)
Pain in extremities	3 (12)	1 (4)	0	0
Hypotension	3 (12)	1 (4)	0	0
Headache	2 (8)	0	2 (11)	0
Muscle spasms	2 (8)	0	2 (11)	0
Musculoskeletal pain	1 (4)	0	3 (17)	0
Prolonged activated partial thromboplastin time	1 (4)	1 (4)	2 (11)	1 (6)
Asthenia	1 (4)	0	2 (11)	0
Cough	1 (4)	0	2 (11)	0
Decreased ejection fraction	1 (4)	0	2 (11)	0
Flank pain	1 (4)	0	2 (11)	1 (6)
Exertional dyspnea	0	0	5 (28)	1 (6)
Urinary tract infection	0	0	4 (22)	0
Lymphopenia	0	0	2 (11)	0
Pyrexia	0	0	2 (11)	0
Thrombocytopenia	0	0	2 (11)	0
Decreased potassium	0	0	2 (11)	0

*AST *aspartate aminotransferase, *TEAE* treatment-emergent adverse event

^a^Includes grouped terms: anemia, hemoglobin decreased

Serious adverse events (SAEs) occurred in 24% (6/25) of patients in the dose escalation portion. All-cause SAEs included disease progression (12% [3/25]), abdominal pain, acute pancreatitis, malignant neoplasm progression, tumor pain, confusional state, renal failure, and hypotension (4% [1/25] each). Of these, the grade 4 confusional state, occurring in a patient receiving the lowest seribantumab dose, 3.2 mg/kg, led to treatment discontinuation. The etiology of the AE was uncertain, and it was considered possibly related to seribantumab due to the time course and therefore scored as a DLT. In this patient, confusion was noticed on Day 13 and on Day 15 the patient was admitted as an in-patient and seribantumab treatment was withheld. Confusion worsened over the following days, then resolved spontaneously on Day 26 without sequelae. No other DLTs were reported in this or other dose cohorts, including the highest dose studied, 40/20 mg/kg.

The MTD was not reached in the dose escalation portion, and the 40/20 mg/kg dose was considered well tolerated and chosen for the dose expansion portion of the study.

#### Dose expansion

In the dose expansion portion of the study, the median duration of exposure was 7 (range, 2‒47) weeks. All patients experienced at least one TEAE and the rate of grade ≥ 3 TEAEs was 50% (9/18) (Table 3). A grade 2 infusion-related reaction was seen in 6% (1/18) of patients. At least one TEAE related to seribantumab occurred in 78% (14/18) of patients. The most common TEAEs related to seribantumab were diarrhea (33%), fatigue (33%), and decreased hemoglobin (28%). An occurrence of grade 3 fatigue related to seribantumab was reported in 6% (1/18) of patients (Table 3).

All-cause SAEs occurred in 44% (8/18) of patients in the dose expansion portion. These included lower abdominal pain, acute pancreatitis, vomiting, disease progression, pyrexia, exertional dyspnea, pleural effusion, arrythmia, dehydration, flank pain, malignant neoplasm progression, hydronephrosis, and ureteric obstruction (6% [1/18] each). None of these events were deemed related to seribantumab.

#### Safety with 40/20 mg/kg dose

All-cause TEAEs in 22 patients receiving the seribantumab 40/20 mg/kg dose across the two study portions are summarized in Online Resource 1. All patients experienced at least one TEAE and the rate of all-cause grade ≥ 3 TEAEs was 45% (10/22). At least one TEAE related to seribantumab occurred in 77% (17/22) of patients. A total of 41% (9/22) of patients reported SAEs; 5% (1/22) of patients had a grade ≥ 3 TEAE related to seribantumab.

### Pharmacokinetics of seribantumab

**Fig. 1:**
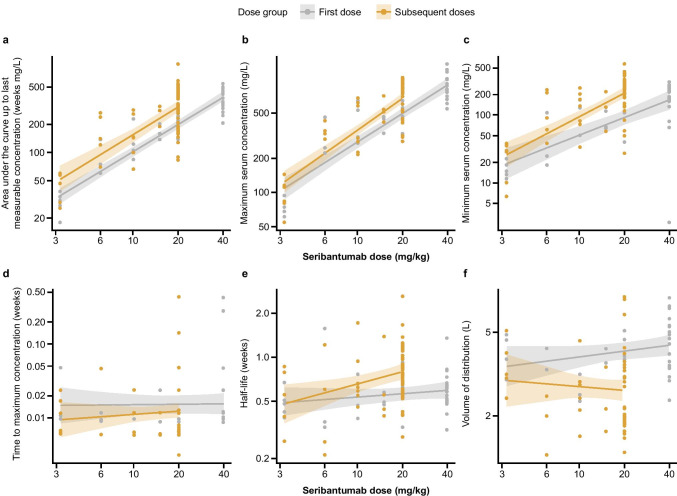
Seribantumab pharmacokinetic parameters by dose. **a** Area under the curve up to last measurable concentration (weeks mg/L). **b** Maximum serum concentration (mg/L). **c** Minimum serum concentration (mg/L). **d** Half-life (weeks). **e** Time to maximum concentration (weeks). **f** Volume of distribution (L)

The PK of seribantumab following the first dose and subsequent doses was analyzed in serum samples (Fig. 1). The area under the curve and serum concentration correlated with seribantumab dose. A dose-dependent increase in area under the curve (0–168 h) and maximum concentration were observed (Fig. 1A‒C). Some accumulation of seribantumab was observed, with the area under the curve accumulation ratio estimated at 1.38 (95% confidence interval [CI]: 1.17‒1.64). Maximum concentration was reached approximately 2 h after the start of infusion and at the end of the infusion (Fig. 1D). The concentration of seribantumab in the blood declined over time with an apparent terminal half-life of 100 h. The half-life for the seribantumab 40/20 mg/kg dose following the second dose was estimated at 5.65 (95% CI: 2.87‒11.27) days. Increasing doses of seribantumab had no effect on half-life or clearance (Fig. 1E). Pre-dose blood concentration during repeated dosing (trough concentration [C_trough_]) increased over time in a dose-dependent manner.

Volume of distribution for the seribantumab 40/20 mg/kg dose following the first dose was estimated at 4.42 L (95% CI: 2.67‒7.32) with no dose dependency observed (Fig. 1F). Following repeated once-weekly infusions of seribantumab, accumulation of seribantumab in the blood was observed and steady state appeared to be achieved by Week 1 Cycle 2, after three to four once-weekly doses had been administered.

In further PK modelling based on historical simulation data, a weight-based dosing of seribantumab 40 mg/kg or 20 mg/kg was determined to correspond to a fixed dose of 3 g or 1.5 g, respectively, when administered on a similar frequency, and was associated with comparable maximum, minimum and average steady state concentrations, and variability. Associations between sex, weight, and seribantumab clearance were found to be significant; however, no significant association was seen between weight and volume.

#### Selection of RP2D

Based on prior preclinical investigations of seribantumab, the maximal inhibition of tumor growth and HER3 is known to be achieved at seribantumab trough concentrations ranging from 100 to 400 mg/L [8]. Specifically, in tumor-bearing mice (A549), treatment with seribantumab 600 µg administered every 3 days, leading to serum trough levels of > 100 mg/L, was associated with up to 125% tumor growth inhibition. Importantly, maximal inhibition and downregulation of HER3 was observed after a single 600 µg dose, confirming the biological activity of this dose level [8].

In the PK analyses of this study, the 40/20 mg/kg dose was shown to result in serum trough concentrations remaining above 100 mg/L in the majority of patients treated (Fig. 1) and the mean C_trough_ was estimated at approximately 200 mg/L. These findings are also consistent with the PK data from a previous Phase 1 study of seribantumab in combination with an oral pan-phosphoinositide 3-kinase (PI3K) inhibitor [10]. Therefore, although no DLTs were observed with the 40/20 mg/kg dose in this study and MTD was not reached, evaluation of seribantumab at higher doses than 40/20 mg/kg was considered unnecessary.

Taken together, the overall tolerability of the 40/20 mg/kg dose combined with supporting PK data from prior preclinical and clinical studies guided the dose selection for the dose expansion portion of the study and, subsequently, confirmed the 40/20 mg/kg dose as the RP2D.

### Efficacy

A majority of patients received at least two cycles of seribantumab treatment, 64% in the dose escalation and 83% in the dose expansion portion (Online Resource 2). At least three treatment cycles were received by 20% and 40% of patients in the dose escalation and dose expansion portions, respectively. One patient in the dose expansion portion received 12 cycles of treatment.

No tumors showed a CR or PR and, therefore, ORR was 0%. Best response of SD was observed in 24% (6/25) of patients in the dose escalation and 39% (7/18) of patients in the dose expansion portion (Table 4). Among the 13 patients who experienced SD across the two study portions, three patients had breast cancer, two had ovarian cancer, two had colorectal cancer, and one patient each had bladder, lung, mucoepidermoid cancer, squamous cell (hip), thymic or urethra carcinoma. Duration of response could not be assessed, as no tumors showed an objective response. In both study portions, 44% of patients experienced progressive disease. Clinical benefit rate, defined as CR, PR, or SD for ≥ 16 weeks, was 0% in the dose escalation portion and 11% (2/18) in the dose expansion portion.

**Table 4:** Best overall response^a^ per RECIST v1.0

Best response	Dose escalation (*n* = 25), n (%)	Dose expansion (*n* = 18), n (%)
Overall response	0	0
Complete response	0	0
Partial response	0	0
Stable disease	6 (24)	7 (39)
Progressive disease	11 (44)	8 (44)

*RECIST v1.0* Response Evaluation Criteria in Solid Tumours version 1.0

^a^Tumor response assessments were not performed for all patients, only those that were reported are shown

Median PFS estimate was 7.1 (95% CI: 4.7‒7.4) weeks in the dose escalation and 7.1 (95% CI: 6.6‒15.9) weeks in the dose expansion portion of the study. The longest median PFS of 14.1 (95% CI: 7.3‒19.1) weeks was observed in the 40/20 mg/kg cohort of the dose escalation. The longest individual PFS observed was 47.9 weeks in a patient with ovarian cancer who had a best response of SD and received 12 cycles of seribantumab treatment.

## Discussion

The objectives of this Phase 1 study were to establish the RP2D and assess the safety, PK, and efficacy of seribantumab monotherapy in patients with advanced or refractory solid tumors. A total of 43 patients with > 10 different tumor types were treated with seribantumab at six dose levels.

Most AEs observed in this study were transient and mild to moderate (grade 1 or 2) in severity. MTD was not reached at the seribantumab dose levels studied and there was no dose dependency to AEs or TEAEs. Only one DLT, grade 4 confusional state that was considered possibly related to seribantumab, occurred with the lowest dose (3.2 mg/kg). Seribantumab 40/20 mg/kg, the highest dose studied, was well tolerated and confirmed as the RP2D in the dose expansion portion of the study, supported by PK findings from prior preclinical and clinical studies [8, 10]. Overall safety findings with this dose were comparable to all other doses. Infusion-related reactions with seribantumab treatment were rare, with only one grade 2 event occurring in the dose expansion portion. Per PK analyses, seribantumab had an apparent terminal half-life of ≈100 h and steady state concentrations were reached after three to four once-weekly doses. No tumors showed a CR or PR; the best response observed was SD, which extended over 16 weeks in a small subset of patients. Taken together, these data support the RP2D of 40/20 mg/kg, equivalent to a fixed dosing regimen of 3 g/1.5 g weekly dosing, in subsequent studies of seribantumab. To date, the safety and tolerability of seribantumab, either as a monotherapy or in combination with other standard anticancer treatments, have been assessed in over 800 patients, demonstrating a consistent safety profile across different regimens and settings. In this context, given that the only DLT reported in this study occurred at the lowest seribantumab dose level and no DLTs were reported in other patients, including those in the highest dose cohort, it can be concluded that this study showed no signal for a trend in DLT.

Targeted therapies have changed the oncology treatment landscape in recent years, moving toward personalized medicine and customized treatment strategies for patients with cancers driven by genomic alterations [11]. Targeting gene fusions in particular increase the potential for therapeutic success; in contrast to gene amplification and overexpression, which are likely to be bystander events, gene fusions are thought to be unique oncogenic drivers [12–14].

Fusions of NRG1, the activating ligand of HER3, are rare genomic alterations found in 0.2%‒0.5% of all solid tumors and have been identified in > 10 unique tumor types [15, 16]. Since the completion of this study, our knowledge of NRG1 fusions and their role in HER3 tumor biology has expanded notably, along with the increasing number of NRG1 fusion partners identified across multiple solid tumor types [12, 17, 18]. Per current understanding, HER3 overactivation by NRG1 fusion proteins represents the primary driver of growth and survival in tumors harboring NRG1 fusions [17–19], and the presence (vs. absence) of NRG1 fusions has been shown to correlate with worsened survival outcomes in lung cancer, including shorter overall survival and disease-free survival [20]. Interestingly, tumors driven by NRG1 gene fusions are unlikely to harbor other known driver alterations such as those in Kirsten rat sarcoma (KRAS) viral oncogene, epidermal growth factor receptor (EGFR), and anaplastic lymphoma kinase (ALK) [12–14].

This expansion of knowledge has led to a rational adjustment in the seribantumab clinical development program, setting focus on solid tumors driven by NRG1 fusions [9, 21]. Data from preclinical studies to date demonstrate encouraging antitumor activity of seribantumab in cancer cell lines and patient-derived xenograft models of various tumor types, including lung, ovarian, and pancreatic tumors harboring different NRG1 fusions and, therefore, support clinical investigations of seribantumab in patients with these types of tumors [9].

When this Phase 1 study was performed, genomic biomarkers were rarely used to guide patient selection for targeted therapy. As a result, the patient population was heterogenous and included patients with tumors refractory to previous treatments, regardless of the primary site or histology. Additionally, although the tumors were initially analyzed for predefined biomarkers (data not reported), including HER3 protein and NRG1 mRNA levels, they were not analyzed for genomic alterations. Subsequent randomized Phase 1 and 2 studies identified detectable NRG1 levels as an important biomarker associated with seribantumab mechanism of action and potential clinical benefit when used in combination with standard of care agents [8, 22], but these studies did not focus on the potential role of seribantumab as monotherapy for patients with tumors harboring NRG1 gene fusions.

The results presented here demonstrate that seribantumab monotherapy is generally well tolerated and associated with manageable AEs across multiple tumor types in a heavily pre-treated patient population. The safety and PK profile of seribantumab described here, combined with the demonstrated preclinical activity in tumor models with NRG1 fusions, support a tumor agnostic strategy for further development of seribantumab in patients with tumors driven by NRG1 fusions. These findings warrant further clinical investigations in these patients who currently have limited treatment options. A Phase 2 study (CRESTONE; NCT04383210) is ongoing and currently enrolling patients with advanced solid tumors harboring NRG1 fusions [21].

## Data Availability

The datasets used and/or analyzed during the current study are available from the corresponding author on reasonable request. Further details are also available via clinical@elevationoncology.com or at https://elevationoncology.com.

## References

[1] Arteaga CL (2014). Engelman JA ERBB receptors: from oncogene discovery to basic science to mechanism-based cancer therapeutics. Cancer Cell.

[2] Dimou A (2019). Camidge DR Detection of NRG1 fusions in solid tumors: rare gold?. Clin Cancer Res.

[3] Trombetta D, Rossi A, Fabrizio FP, Sparaneo A, Graziano P, Fazio VM (2017). NRG1-ErbB lost in translation: a new paradigm for lung cancer?. Curr Med Chem.

[4] Lee-Hoeflich ST, Crocker L, Yao E, Pham T, Munroe X, Hoeflich KP (2008). A central role for HER3 in HER2-amplified breast cancer: implications for targeted therapy. Can Res.

[5] Beji A, Horst D, Engel J, Kirchner T (2012). Ullrich A Toward the prognostic significance and therapeutic potential of HER3 receptor tyrosine kinase in human colon cancer. Clin Cancer Res.

[6] Chung YW, Kim S, Hong JH, Lee JK, Lee NW, Lee YS (2019). Overexpression of HER2/HER3 and clinical feature of ovarian cancer. Journal of Gynecologic Oncology.

[7] Lyu H, Han A, Polsdofer E, Liu S, Liu B (2018). Understanding the biology of HER3 receptor as a therapeutic target in human cancer. Acta Pharm Sin B.

[8] Schoeberl B, Kudla A, Masson K, Kalra A, Curley M, Finn G (2017). Systems biology driving drug development: from design to the clinical testing of the anti-ErbB3 antibody seribantumab (MM-121). NPJ Systems Biology and Applications.

[9] Odintsov I, Lui AJW, Sisso WJ, Gladstone E, Liu Z, Delasos L (2021). The Anti-HER3 mAb Seribantumab Effectively Inhibits Growth of Patient-Derived and Isogenic Cell Line and Xenograft Models with Oncogenic NRG1 Fusions. Clin Cancer Res.

[10] Abramson VG, Supko JG, Ballinger T, Cleary JM, Hilton JF, Tolaney SM (2017). Phase Ib study of safety and pharmacokinetics of the PI3K inhibitor SAR245408 with the HER3-neutralizing human antibody SAR256212 in patients with solid tumors. Clin Cancer Res.

[11] Garinet S, Laurent-Puig P, Blons H (2018). Oudart JB Current and future molecular testing in NSCLC, what can we expect from new sequencing technologies?. J Clin Med.

[12] Fernandez-Cuesta L, Plenker D, Osada H, Sun R, Menon R, Leenders F (2014). CD74-NRG1 fusions in lung adenocarcinoma. Cancer Discov.

[13] Drilon A, Somwar R, Mangatt BP, Edgren H, Desmeules P, Ruusulehto A (2018). Response to ERBB3-directed targeted therapy in NRG1-rearranged cancers. Cancer Discov.

[14] Jones MR, Williamson LM, Topham JT, Lee MKC, Goytain A, Ho J (2019). NRG1 gene fusions are recurrent, clinically actionable gene rearrangements in KRAS wild-type pancreatic ductal adenocarcinoma. Clin Cancer Res.

[15] Russo A, Lopes AR, Scilla K, Mehra R, Adamo V, Oliveira J et al (2020) NTRK and NRG1 gene fusions in advanced non-small cell lung cancer (NSCLC). Precision Cancer Med 3(14). 10.21037/pcm.2020.03.02

[16] Stalbovskaya V, Wasserman E, Fryzek J, Bylsma LC, Sirulnik LA (2020) NRG1 fusion-driven cancers: a systematic literature review and meta-analysis. J Clin Oncol 38(15_suppl):e15605-e. 10.1200/JCO.2020.38.15_suppl.e15605

[17] Jonna S, Feldman RA, Swensen J, Gatalica Z, Korn WM, Borghaei H (2019). Detection of NRG1 gene fusions in solid tumors. Clin Cancer Res.

[18] Laskin J, Liu SV, Tolba K, Heining C, Schlenk RF, Cheema P (2020). NRG1 fusion-driven tumors: biology, detection, and the therapeutic role of afatinib and other ErbB-targeting agents. Ann Oncol.

[19] Fernandez-Cuesta L (2015). Thomas RK Molecular pathways: targeting NRG1 fusions in lung cancer. Clin Cancer Res.

[20] Shin DH, Lee D, Hong DW, Hong SH, Hwang JA, Lee BI et al (2016) Oncogenic function and clinical implications of SLC3A2-NRG1 fusion in invasive mucinous adenocarcinoma of the lung. Oncotarget 7(43):69450–69465. 10.18632/oncotarget.11913 10.18632/oncotarget.11913 PMC5342490 27626312

[21] Bendell JC, Lim K-H, Burkard ME, Lin JJ, Chae YK, Socinski MA et al (2020) Abstract PO-003: CRESTONE – Clinical study of response to seribantumab in tumors with neuregulin-1 (NRG1) Fusions – A phase 2 study of the anti-HER3 mAb for advanced or metastatic solid tumors (NCT04383210). Cancer Res 80(22_suppl):PO-003-PO. 10.1158/1538-7445.PANCA20-PO-003

[22] Liu JF, Ray-Coquard I, Selle F, Poveda AM, Cibula D, Hirte H (2016). Randomized phase II trial of seribantumab in combination with paclitaxel in patients with advanced platinum-resistant or -refractory ovarian cancer. J Clin Oncol.

